# Commensal *Escherichia coli* inhibits the growth and modulates the fitness, virulence, and antimicrobial resistance of *Salmonella* Heidelberg *in vitro*

**DOI:** 10.1128/spectrum.03336-24

**Published:** 2025-08-18

**Authors:** Yasir R. Khan, Lekshmi K. Edison, Thomas Denagamage, Subhashinie Kariyawasam

**Affiliations:** 1 Department of Large Animal Clinical Sciences, College of Veterinary Medicine, University of Florida 374692, Gainesville, Florida, USA; 2 Department of Comparative, Diagnostic, and Population Medicine, College of Veterinary Medicine, University of Florida 3463, Gainesville, Florida, USA; The Ohio State University College of Dentistry, Columbus, Ohio, USA

**Keywords:** antibiotic resistance, commensal *Escherichia coli*, fitness, food safety, probiotics, *Salmonella *Heidelberg, virulence

## Abstract

Nontyphoidal *Salmonella* (NTS) are major foodborne pathogens primarily transmitted to humans through contaminated poultry products. Increased antimicrobial resistance (AMR) in NTS, including *Salmonella* Heidelberg (SH), has recently become a public health issue. Current control measures are inadequate, emphasizing the need for novel approaches to mitigate NTS colonization in poultry and contamination of poultry products. We hypothesized that commensal *Escherichia coli* can reduce antibiotic-resistant NTS colonization in the chicken intestines by modulating the fitness, virulence, and AMR potential of *Salmonella*. To test this hypothesis, we co-cultured a commensal *E. coli* strain (EC47-1826) isolated from a commercial broiler chicken and an antibiotic-resistant SH strain (SH18-9079) isolated from the liver of a turkey and analyzed their transcriptomes using RNA-sequencing. Our analysis revealed 4,890 differentially expressed genes in SH when co-cultured with commensal *E. coli*. After filtering the expression data, we found 193 genes were significantly upregulated, while 202 genes were downregulated. Notably, several genes involved in bacterial growth, pathogenicity and virulence, biofilm formation, metal-ion homeostasis, signal transduction and chemotaxis, stress response, transmembrane transport of xenobiotics, and cellular metabolism were downregulated by as much as 86-folds in SH as compared to the control. Furthermore, this study revealed the downregulation of genes associated with AMR and drug efflux in SH by up to 12 folds. These findings highlight that commensal *E*. c*oli* may reduce the fitness, persistence, virulence, and AMR dissemination of SH, implying that *E. coli* strains could be utilized to mitigate antibiotic-resistant SH in poultry, ultimately enhancing food safety.

**IMPORTANCE:**

NTS, commonly transmitted to humans through contaminated poultry meat and eggs, is a frequent cause of foodborne illness. Augmenting the situation, foodborne outbreaks of antibiotic-resistant NTS have become an additional food safety and public health concern. Evaluation of growth changes and transcriptomic profiling of antibiotic-resistant SH and commensal *E. coli* in a mixed culture of the two will provide insights into the ability of commensal *E. coli* to reduce SH colonization of chicken intestines and the genes involved in that change. Our study showed that commensal *E. coli* significantly reduced antibiotic-resistant SH counts and expression of *Salmonella* genes, which play a vital role in their growth and persistence. This suggests the potential use of commensal *E. coli* to control antibiotic-resistant SH colonization in poultry, leading to improved food safety through reduced NTS contamination of foods of poultry origin and reduced dissemination of antibiotic-resistant *Salmonella* to humans via the food chain.

## INTRODUCTION

Nontyphoidal *Salmonella* (NTS) is a frequent cause of bacterial foodborne illness in humans globally (1). Among the various NTS serovars, *Salmonella enterica* serovar Heidelberg (SH) is notably recognized as a serovar associated with foodborne illness and exhibits resistance to antibiotics critical to human health (2–6). As a matter of concern, there has also been a significant increase in multidrug resistance (MDR) in SH isolates over the last two decades (5). The National Antimicrobial Resistance Monitoring System reported that approximately one-fifth of human isolates of SH in 2021 in the United States exhibited MDR (1). The SH is frequently isolated from food products of animal origin, and poultry is considered one of the primary reservoirs of antibiotic-resistant NTS and their resistance gene repertoire (5). The more invasive and severe forms of SH infections in humans compared to other *Salmonella* serovars indicate their presence in poultry production systems as a critical problem due to the increased risk of contamination of poultry commodities and subsequent transmission to humans (7). Furthermore, owing to the ease of dissemination and continuous increase in virulence, the spread of SH, mainly through poultry products, is considered a significant public health and food safety issue (8).

Many countries, particularly in the developing world, continue to use antibiotics to control *Salmonella* in poultry, raising public health concerns due to its potential contribution to AMR in *Salmonella* serovars (9). The emergence of MDR in SH, especially against extended-spectrum cephalosporins (ESCs), has significantly reduced the treatment options for invasive SH infections in humans (3, 5). Therefore, various antibiotic alternative strategies for NTS control are implemented at pre- and post-harvest levels. Pre-harvest strategies include utilizing non-antibiotic products such as probiotics, prebiotics, synbiotics, postbiotics, and bacteriophages along with farm biosecurity measures and routine *Salmonella* vaccine programs. At the post-harvest level, sanitary protocols, hygiene measures, and carcass treatment practices are enforced in processing plants (10, 11). However, recent multistate outbreaks linked to SH in poultry highlight inconsistent outcomes and limited effectiveness of these pre-harvest and post-harvest approaches to minimize *Salmonella* colonization and persistence in the poultry intestines, their dissemination in the environment, and contamination of the food chain emphasizing the need for more effective and reliable interventions across the poultry production continuum (8, 10–16). Among the variuos approches being investigated, developing non-toxic, residue-free, potent agents to control *Salmonella* colonization in poultry has been the focus of contemporary research efforts. Such microbial agents, called probiotics, are being considered because of their effectiveness against pathogen colonization in the intestines as well as additional beneficial effects on the host (17).

Probiotics have been shown to enhance resistance to *Salmonella* colonization in the poultry gut (12). In this context, commensal *E. coli* can play a crucial role in effectively controlling the intestinal colonization of *Salmonella* in chickens (18). Commensal *E. coli* living symbiotically in the chicken gut are involved in several functions, including intestinal cell maturation and development, enzyme production, nutrient synthesis, immunomodulation, homeostasis, competitive exclusion of pathogens, and modulation of the intestinal microbiome (19). Since intestinal commensal *E. coli* are known to co-exist with *Salmonella* in the intestinal tract of chickens, efforts are underway to identify specific strains of commensal *E. coli* as probiotics against NTS serovars in poultry (16, 20, 21). However, the effects of intestinal commensal *E. coli* of poultry origin on the colonization, persistence, virulence, and AMR dissemination of foodborne antibiotic-resistant SH in the chicken intestinal tract has not yet been fully elucidated.

Transcriptomic profiling provides global gene expression patterns central to understanding the complex interplay between microbial populations. Comparative transcriptomic analysis further identifies differentially expressed genes and pathways under specific conditions, providing a holistic insight into the molecular basis for interactions between the host and pathogen or among the members of different microbial communities (22). However, literature describing the comparative transcriptomic profiling of SH in the presence of commensal *E. coli* remains scarce. Therefore, to narrow this knowledge gap, the current study examined the transcriptomic profile of an antibiotic-resistant strain of SH in the presence of an intestinal commensal strain of *E. coli* derived from poultry. Here, we hypothesized that the intestinal commensal *E. coli* of poultry origin could reduce the expression of genes critical for the fitness, virulence, and dissemination of AMR in the antibiotic-resistant SH strain. Through whole-genome transcriptomic profiling, we investigated the differential gene expression of SH in the presence of commensal *E. coli* and explored the effects of commensal *E. coli* on the fitness, virulence, and AMR dissemination potential of SH to use it as a pre-harvest intervention strategy to mitigate the impacts of intestinal NTS colonization in poultry.

## RESULTS

### Transcriptomic profile of SH and commensal *E. coli* in co-culture

**Fig 1:**
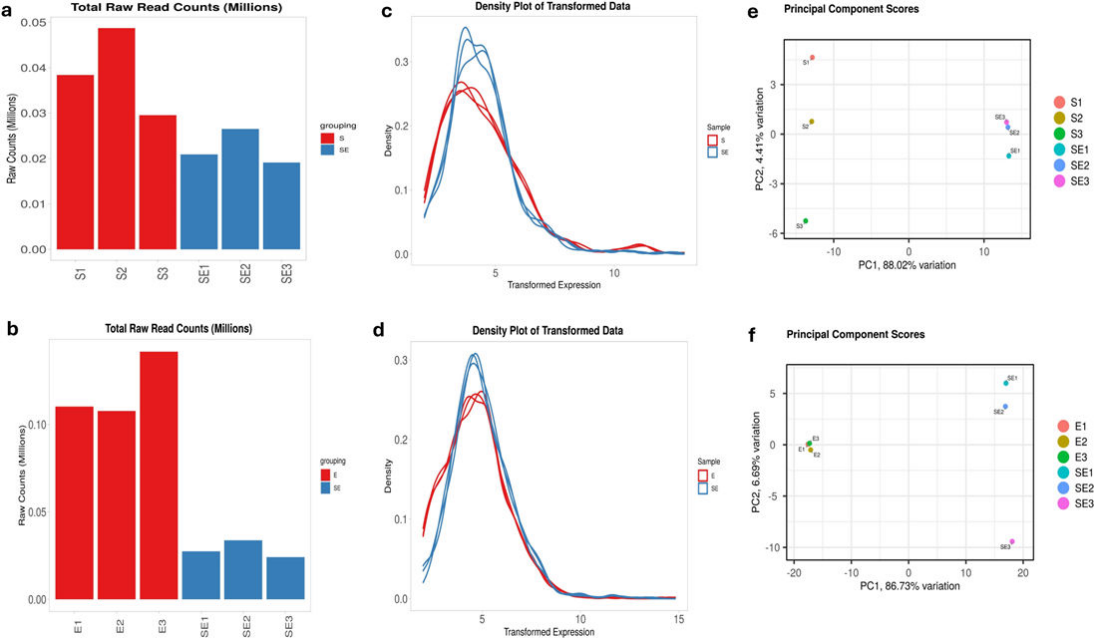
Diagnostic plots illustrating the sequencing depth bias for *S*. Heidelberg strain SH18-9079 and commensal *E. coli* strain EC47-1826 grown alone (controls) and in co-culture. Total raw read count per library (**a, b**), density plot of regularized log-transformed total read counts (**c, d**), and principal component analysis (PCA) (**e, f**) of *S*. Heidelberg 18-9079 and commensal EC47-1826, respectively. The difference between the SH18-9079 (**S1, S2, S3**) grown alone (control) and SH18-9079 in co-culture with EC47-1826 (SE1, SE2, and SE3) accounted for the observed 88.02% variation in SH18-9079 in the co-culture compared to the control (**Fig. 2e**). The total read counts differed significantly among the sample groups (*P* = 4.91e-02), based on ANOVA. Total read counts differed significantly among sample groups (*P* = 1.26e-03) based on ANOVA.

The transcript reads were mapped to *Salmonella enterica* subsp. *enterica* serovar Heidelberg (GCF_016452005.1) and ESC-resistance encoding plasmid (NZ_MW 349106.1) for SH and E. coli APECO1 (GCF_000014845.1) for commensal *E. coli*. The percentage map-read coverages of transcript reads were 99.64 and 99.31 for SH and *E. coli* grown individually (control) and 99.47 and 99.52 for SH and *E. coli* in the co-culture, respectively. Diagnostic plots for the read count data were generated to analyze the variations in mapped library sizes of significantly differentially expressed genes (SDEGs) for SH (Fig. 1a, c and e) and commensal *E. coli* (Fig. 1b, d and f).

### Overall transcriptomic differences indicated downregulation of SH genes responsible for propagation, persistence, virulence, and antibiotic resistance dissemination

**Fig 2:**
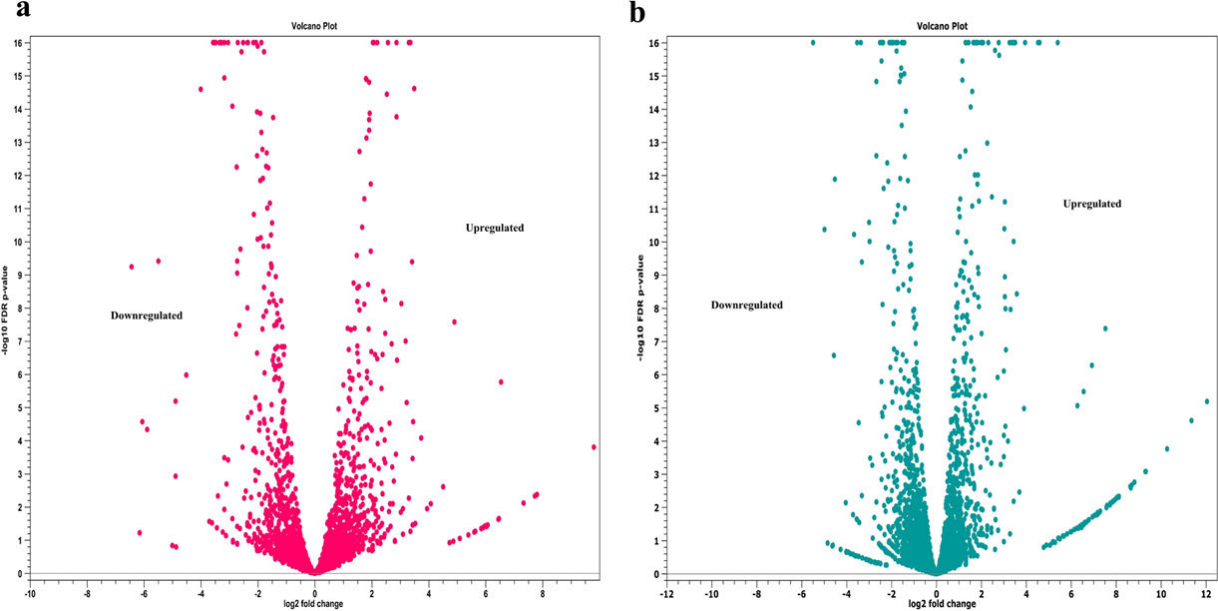
Volcano plots depicting the differential transcriptomic profile of *S*. Heidelberg strain SH18-9079 (a) and commensal *E. coli* strain EC47-1826 (b) in co-culture. This figure provides the relationship between the magnitude (X axis = log_2_ fold change) and extent of gene expression (Y axis = *P*-value) in the overall transcriptomic expression of SH18-9079 and commensal EC47-1826, respectively.

**Fig 3:**
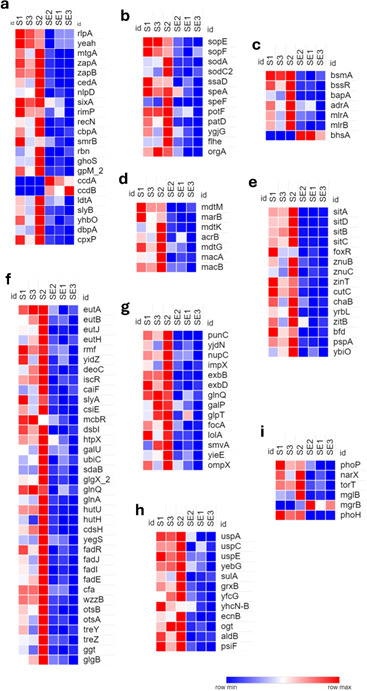
Heatmaps of transcriptional responses of *S*. Heidelberg strain SH18-9079 when co-cultured with commensal *E. coli* strain EC47-1826 based on functional analysis. (a) Cell proliferation, (b) pathogenicity and virulence factors, (c) biofilm formation, (d) antimicrobial resistance and multidrug efflux, (e) ion homeostasis, (f) cellular metabolism, (g) outer membrane proteins and transmembrane transporters, (h) stress response regulation, and (i) signal transduction and chemotaxis. The transformed Fragments/Kilobase of Transcript/Million (FPKM) numbers (normalized read counts) are illustrated in color. Blue indicates low expression, whereas red indicates high expression.

The transcriptomics profile of SH co-cultured with commensal *E. coli* differed from that of SH grown alone, and gene expression analysis showed a significant downregulation of genes critical for SH propagation, persistence, virulence, and AMR dissemination in the presence of commensal *E. coli*. Results of the overall differential gene expression analysis of SH when co-cultured with *E. coli* (SH *+E. coli* vs SH alone as the control) are presented in Table S2 (Table S2). Briefly, among 4,968 differentially expressed genes (DEGs) in SH when co-cultured with *E. coli,* 2,158 genes were downregulated and 2,230 were upregulated, whereas 580 hypothetical genes exhibited no detectable change. However, after applying gene filtration criteria, only 395 of 4,968 genes remained as SDEGs in SH. Of these 395 SDEGs of SH, 193 genes were downregulated and 202 were upregulated. Overall differential transcriptomic changes in SH are presented in volcano plots (Fig. 2a), and heatmaps of the expression profiles of different SDEG categories are presented in Fig. 3.

### Overall transcriptomic differences showed upregulation of *E. coli* genes responsible for propagation, adherence and motility, metal ion homeostasis, stress response regulation, and signal transduction

**Fig 4:**
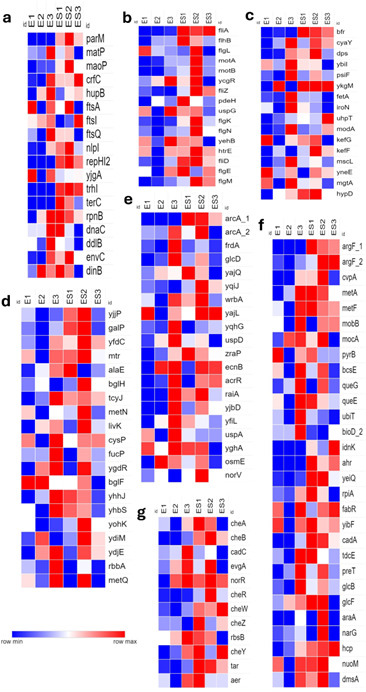
Heatmaps of transcriptional responses of commensal *E. coli* strain EC47-1826 in co-culture with *S*. Heidelberg strain SH18-9079 based on functional analysis. (a) Cell proliferation, (b) adherence and bacterial motility, (c) ion homeostasis and regulation, (d) outer membrane proteins and transmembrane transporters, (e) stress response regulation, (f) cellular metabolism, and (g) signal transduction and chemotaxis. The transformed Fragments/Kilobase of Transcript/Million (FPKM) numbers (normalized read counts) are illustrated in color. Blue indicates low expression, whereas red indicates high expression.

A significant (*P*-value ≤ 0.01) upregulation of genes important for propagation, adherence and bacterial motility, ion homeostasis, signal transduction and chemotaxis, stress management, transmembrane transportation, and cellular metabolism was observed in *E. coli* on gene expression analysis in the presence of antibiotic-resistant SH. Briefly, among 5,227 DEGs in *E. coli* in co-culture with SH*,* 2,023 genes were upregulated, 2,021 genes were downregulated whereas 1,183 hypothetical genes exhibited no detectable change. However, 660 genes were SDEGs out of 5,227 genes in *E. coli* after the implementation of gene filtration criteria. Of these 660 SDEGs**,** 237 genes were upregulated and 423 were downregulated. Overall differential transcriptomic changes in *E. coli* are presented in the volcano plot (Fig. 2b). Heatmaps of the expression profiles of different SDEG categories are presented in Fig. 4. Results of the overall differential gene expression analysis of *E. coli* when co-cultured with SH (SH *+ E. coli* vs *E. coli* alone as the control) are presented in Table S3 (Table S3).

### Enriched biological pathways of SH in co-culture with commensal *E. coli*

**Fig 5:**
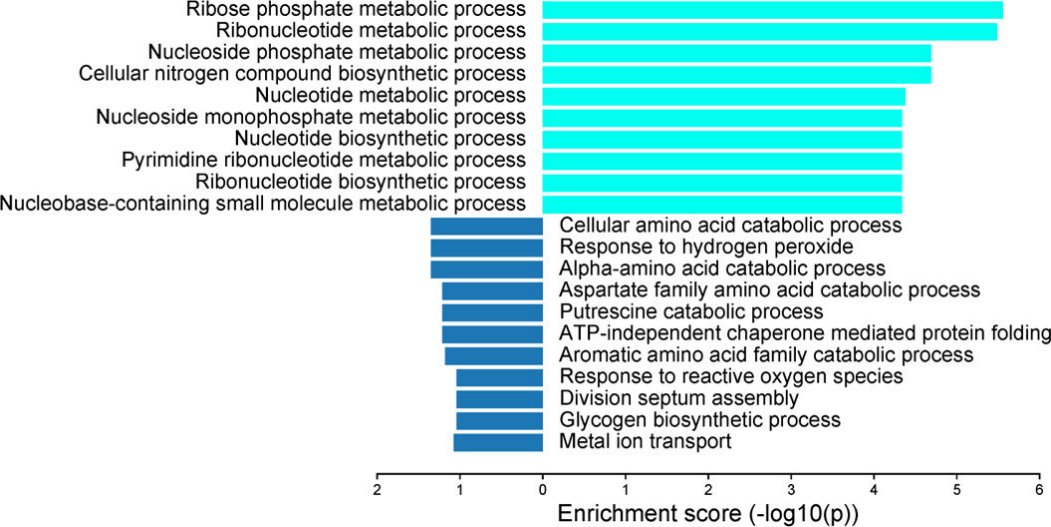
Enriched biological pathways of SDEGs in *S*. Heidelberg strain SH18-9079 in the presence of commensal *E. coli* strain EC47-1826. The X-axis shows the enrichment score (-log 10 *P*-value).

Gene enrichment analysis categorized the SDEGs according to their respective biological pathways and metabolic functions to highlight the molecular and cellular processes involved in the interactions between SH and *E. coli* when they were co-cultured. Briefly, in SH, a significant downregulation was observed for the processes involved in cell division septum assembly formation, glycogen biosynthesis, metal ion transport, putrescine catabolism, response to reactive oxygen species and hydrogen peroxide , ATP-independent protein folding, and amino acid catabolism. In contrast, biological processes involved in the metabolism of ribose phosphate, ribonucleotides, nucleoside phosphate, nucleotides, pyrimidine ribonucleotides, nucleoside-monophosphate and nucleobase-containing small molecules, and biosynthesis of cellular nitrogen compounds, nucleotides and ribonucleotides remained upregulated in SH in the presence of *E. coli* (Fig. 5).

### Enriched biological pathways of commensal *E. coli* in co-culture with SH

**Fig 6:**
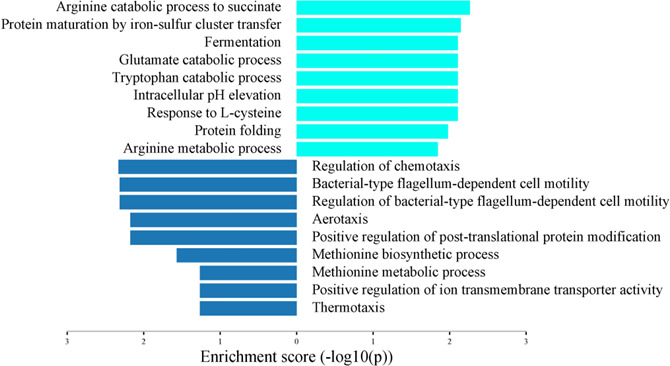
Enriched biological pathways of SDEGs in commensal *E. coli* strain EC47-1826 in the presence of *S*. Heidelberg strain SH18-9079. The X-axis shows the enrichment score (-log 10 *P*-value).

In *E. coli*, gene enrichment analysis of SDEGs revealed a significant upregulation of the molecular and cellular processes especially involved in the arginine metabolism, arginine catabolism to glutamate and succinate, maturation of protein by iron-sulfur cluster transfer, fermentation, catabolism of glutamate and tryptophan, elevation of intracellular pH, response to L-cysteine, and protein folding. In contrast, biological processes involved in chemotaxis, flagellum-dependent cell motility, aerotaxis, methionine biosynthesis and metabolism, positive regulation of ion transmembrane transporter activity, and thermotaxis were downregulated in *E. coli* in the presence of SH (Fig. 6).

### Bacterial growth patterns

**Fig 7:**
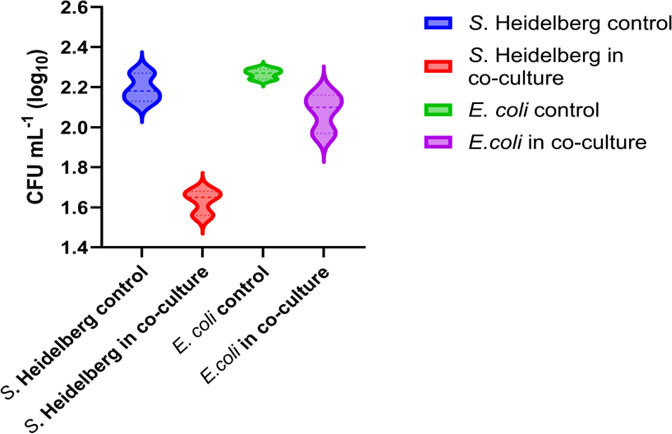
Colony-forming units (mean ± SD × log_10_ CFU/mL) of *S*. Heidelberg SH18-9079 and commensal *E. coli* EC47-1826 in co-culture and controls. The log_10_ CFU/mL of *S*. Heidelberg in the co-culture was significantly less than that of the control (*P* ≤ 0.05).

When an equal number of cells (2.66 × 10^7^ CFU/mL) of each SH and commensal *E. coli* were co-cultured for 24 h, the viable colony count of SH (1.63 ± 0.06 log_10_ CFU/mL) in the co-culture was significantly less than that of the *Salmonella* grown alone (2.2 0 ± 0.07 log_10_ CFU/mL) (*P* = 0.017). Similarly, the colony count of SH (1.63 ± 0.06 log_10_ CFU/mL) in the co-culture was significantly less than that of the commensal *E. coli* (2.08 ± 0.10 log_10_ CFU/mL) (*P* = 0.003). However, the difference observed between the colony counts of commensal *E. coli* (2.27 ± 0.03 log_10_ CFU/mL) grown alone and in co-culture (2.08 ± 0.10 log_10_ CFU/mL) (*P* = 0.1013) was statistically insignificant (Fig. 7).

### Functional annotation of significantly differentially expressed genes of SH when co-cultured with commensal *E. coli*

#### Cell proliferation

The results demonstrated a significantly low expression of SH genes involved in cell proliferation in the presence of commensal *E. coli,* which aligned with the reduced *Salmonella* colony counts (CFU/mL) recorded during the experiment (Fig. 7). Specifically, twenty genes, including five genes associated with cell wall organization, cell shape regulation, and cell wall biogenesis (*rlpA, yeaH, mtgA, idtA,* and *sylB)*; four genes involved in RNA/DNA endonuclease activity (*smrB, rbn, ghoS,* and *gpM-2*); two genes each for cell septum assembly formation during bacterial cell division (z*apA* and *zapB*), cell division and modulation (*cedA* and *nlpD),* DNA repair (*recN* and *yhbO)*; and one gene each for protein modification (*sixA*), small ribosomal biogenesis (*rimP*), protein folding (*cbpA*), RNA helicase activity (*dbpA*), and cell envelope modulation (*cpxP*), were downregulated in SH. Contrary to this observation, two genes associated with the type II toxin-antitoxin system (*ccdA* and *ccdB*) in SH were upregulated (Fig. 3a).

#### Pathogenicity and virulence factors

Twelve genes involved in the pathogenicity and virulence of *Salmonella* were downregulated in SH. Particularly, the genes involved in the regulation of type III secretion system (T3SS) effectors (*sopE* and *sopF*), superoxide dismutase (*sodC2* and *sodA*), and secretion system apparatus proteins (*ssaD*) were downregulated. Additionally, genes involved in the biosynthesis of polyamines, such as the putrescine precursor/arginine decarboxylase (*speA*), ornithine decarboxylase (*speF*), putrescine transporter (*potF*), and putrescine catabolism enzymes (*patD* and *ygjG*), were downregulated. Furthermore, there was notable downregulation of genes related to flagellar protein biosynthesis (*flhE*) and those involved in the flagellar biosynthesis/type III secretory pathway (*orgA*) (Fig. 3b).

#### Biofilm formation

This study exhibited the expression of genes vital for biofilm formation and regulation in *Salmonella*. A significant downregulation was observed for four genes (*bsmA, bssR, adrA,* and *bapA*) associated with biofilm formation, as well as for two genes (*mlrA* and *mlrB*) that play a role in *csgD* transcription, which is the master regulator of biofilm formation and cellulose biosynthesis. Conversely, an upregulation was observed for *bhsA*, involved in reduction of biofilm formation (Fig. 3c).

#### Antimicrobial resistance and multidrug efflux

In this study, several genes in SH implicated in AMR and multidrug efflux were downregulated significantly in the presence of commensal *E. coli*. These included the genes that encode an MDR transporter (*mdtM*), multiple antibiotic resistance protein (*marB*), macrolide transporter subunit and macrolide ABC transporter ATP-binding proteins/permeases (*macA* and *macB*), multidrug efflux and toxic compound extrusion (MATE) transporter activation protein (*mdtK*), other multidrug efflux pump activation proteins (*acrB* and *mdtG*), and a protein involved in the regulation of the proton-dependent efflux pump (*mdtM*) (Fig. 3d). Furthermore, the extended-spectrum beta-lactamase (ESBL) gene *bla*_CTX-M-1_ was also downregulated in SH when it was co-cultured with commensal avian *E. coli*.

#### Ion homeostasis

Fifteen genes associated with metal ion acquisition, utilization, transportation, and storage were downregulated in SH. Specifically, five genes involved in iron transport and uptake (*sitA, sitB, sitC, sitD,* and *foxR*)*,* three in zinc-ion transmembrane transport (*znuB, znuC,* and *zitB*)*,* and one each in the iron mobilization/bacterioferritin-associated ferredoxin (*bfd*)*,* zinc-ion binding (*ZinT*), copper homeostasis (*cutC*)*,* cation transportation (*chaB*)*,* and magnesium-ion regulation (*yrbL*) demonstrated significant downregulation in SH in addition to the genes involved in divalent metal acquisition and transport (*pspA*) and monoatomic ion channel activity (*ybiO*) (Fig. 3e).

#### Cell metabolism

Several genes involved in critical cellular metabolic processes of SH also demonstrated significant downregulation. These included the genes involved in the catabolism and transport of ethanolamine (*eutA, eutB, eutH,* and *eutJ*); protein biosynthesis and catabolism (*rmf, yidZ, deoC, iscR, caiF, slyA, csiE, mcbR*, *dsbI,* and *htpx*); carbohydrate biosynthesis and gluconeogenesis (*galU, ubiC, sdaB,* and *glgX-2*); amino acid metabolism (*glnQ*, *glnA, hutU, hutH,* and *cdsH)*; lipid, lipopolysaccharide, and fatty acid metabolism (*yegS, fadR, fadJ, fadI, fadE, cfa,* and *wzzB*); trehalose biosynthesis (*otsA, otsB, treY,* and *treZ*); glutathione degradation (*ggt*); and glycan biosynthesis (*glgB*) (Fig. 3f).

#### Outer membrane proteins and transmembrane transporters

The expression of genes associated with certain outer membrane proteins (OMPs), such as OmpX and transmembrane transport of xenobiotics (*punC*), metalloproteins (*yjdN*), nucleosides (*nupC* and *impX*), proteins and amino acids (*exbB, exbD*, and *glnQ*), sugars (*galP* and *glpT*), formate (*focA*), lipoprotein localization to the outer membrane (*lolA*), and coenzyme transport and metabolism (*yieE*) remained notably downregulated. We also observed a considerably low expression of *smvA*, a gene involved in the transmembrane transportation of acriflavine and other quaternary ammonium compounds (Fig. 3g).

#### Stress response regulation

The expression of genes responsible for stress response mechanisms in SH showed significant downregulation. These downregulated genes are specifically involved in the universal stress response (*uspA, uspC,* and *uspE*), SOS response (*yebG* and *sulA*), glutathione activity (*grxB* and *yfcG*), peroxide damage (*yhcN-B*), response to toxic substances (*ecnB*), cellular responses to DNA protection/repair (*ogt*), aldehyde dehydrogenation to mitigate the oxidative/electrophilic stresses (*aldB*), and oxidative stress/phosphorus starvation stress (*psiF*) (Fig. 3h).

#### Signal transduction and chemotaxis

Several genes involved in signaling and chemotaxis in SH, particularly those associated with phosphorelay signal transduction and predominantly responding to low levels of external divalent cations zinc and magnesium (*phoP*), phosphorelay sensory kinase/sensory histidine kinase (*narX*), phosphate regulator induced by phosphate starvation (*phoH),* periplasmic sensors of multicomponent regulatory systems/response regulators in the Tor respiratory system (*torT*), and chemotaxis (*mglB*) were downregulated. In contrast, *mgrB*, a negative regulator of the bacterial phosphorelay signal transduction system, was upregulated (Fig. 3i).

### Functional annotation of significantly differentially expressed genes of commensal *E. coli* when co-cultured with SH

#### Cell proliferation

Our results showed a significantly high expression of *E. coli* genes involved in cell proliferation in the presence of *Salmonella,* consistent with the observed increase in *E. coli* colony counts during the experiment (Fig. 7). These 18 upregulated *E. coli* genes included five genes associated with chromosome segregation, organization, and condensation (*parM*, *matP*, *moaP*, *crfC*, and *hupB*), three genes involved in the barrier septum assembly formation (*ftsA, ftsI,* and *ftsQ*), three genes for cell division (*nlpl*, *repHI2,* and *yjgA*), and one gene each involved in 3-5′ DNA helicase activity and unwinding of duplex DNA (*trhI*), protection from abnormal sticking together or degradation of chromosomes (*terC*), double-stranded DNA endonuclease activity and DNA recombination (*rpnB*), elongation of DNA strands during DNA replication (*dnaC*), cell shape regulation (*ddIB*), cell separation after cytokinesis (*envC*), and DNA-dependent DNA replication (*dinB*) (Fig. 4a).

#### Adherence and bacterial motility

The results indicated an upregulation of 16 genes critical for the adherence and bacterial motility in *E. coli* in the co-culture. These included 14 genes responsible for bacterial-type flagellum-dependent cell motility, including their regulation and assembly (*fliA*, *flhB, flgL, motA, motB, ycgR, fliZ, pdeH, uspG, flgK, flgN, fliD, flgE* and *flg*M), and two genes for pilus assembly formation and cell adhesion (*yehB* and *htrE*) (Fig. 4b).

#### Ion homeostasis

In this study, 17 genes associated with metal ion acquisition, storage, and homeostasis were upregulated in commensal *E. coli* grown together with SH. Particularly, the genes responsible for intracellular sequestration of iron (*bfr*), iron-sulfur cluster assembly formation (*cyaY*), cellular iron homeostasis (*dps*), iron binding (*hypD*), zinc binding (*ybiI*), cellular response to phosphate starvation (*psiF*), and cellular response to zinc starvation (*ykgM*) were upregulated. Genes associated with transmembrane movement and transportation of iron (*fetA*), siderophores (*iroN*), phosphate (*uhpT*), molybdate (*modA*), potassium (*kefG* and *kefF*), monoatomic-ions (*mscL*), chloride (*yneE*), and magnesium (*mgtA*) were also significantly upregulated in the commensal *E. coli* in co-culture with SH (Fig. 4c).

#### Outer membrane proteins and transmembrane transporters

The expressions of genes associated with OMPs and vital for transmembrane transport of succinate (*yjjP*), galactose (*galP*), formate (*yfdC*), amino acids (*mtr* and *alaE*), polysaccharides (*bglH*), cysteine (*tcyJ*), D-methionine (*metN* and *metQ*), leucine (*livK*), thiosulfate (*cysP*), fucose (*fucP*), histidine (*ygdR*), and trehalose (*bglF*) were upregulated in *E. coli*. Furthermore, genes involved in ABC-type transportation activity (*rbbA*, *yhhJ*, and *yhbS*) and 3-hydroxypropanoate export (*yohK*) were also significantly upregulated. We also noted the upregulation of genes (*ydiM* and *ydjE*) associated with the major facilitator superfamily (MFS) type transmembrane transport activity (required for cellular Fe and Cu homeostasis for the biogenesis of different heme-Cu oxygen reductases) in the commensal *E. coli* (Fig. 4d).

#### Stress response regulation

Twenty genes responsible for stress response mechanisms in commensal *E. coli* were upregulated. Importantly, genes involved in aerobic respiration control (*arcA-1* and *arcA-2*), cellular response to DNA damage stimulus (*frdA, glcD, yajQ,* and *yqiJ*), oxidative stress (*wrbA*, *yajL*, and *yqhG*), superoxides (*uspD*), cellular response to cell envelope stress (*zraP*), toxic substances (*ecnB*), and xenobiotic stimulus (*acrR*) were upregulated. Furthermore, genes associated with the bacterial dormancy process in unfavorable conditions (*raiA*), general and universal stress responses (*yjbD, yfiL*, and *uspA*), osmotic stress (*yghA* and *osmE*), and response to nitric oxide (*norV*) were also notably upregulated in commensal *E. coli* in the presence of SH (Fig. 4e).

#### Cell metabolism

Several genes involved in critical cellular metabolic processes were significantly upregulated in *E. coli*. These genes included those associated with the biosynthesis of arginine (*argF-1* and *argF-2*), toxin production (*cvpA*), methionine synthesis (*metA* and *metF*), Mo-molybdopterin cofactor biosynthetic process (*mobB* and *mocA*), pyrimidine metabolism (*pyrB*), cellulose production (*bcsE*), queuosine synthesis (*queG* and *queE*), ubiquinone production (*ubiT*), and biotin synthesis (*bioD-2*). Similarly, genes involved in the metabolism of D-gluconate (*idnK* and *ahr*), mannitol (*yeiQ*), ribose (*rpiA*), fatty acids (*fabR*), and glutathione (*yibF*) were also upregulated. Furthermore, several other genes responsible for catabolism, particularly those related to lysine (*cadA*), L-threonine to propionate (*tdcE*), thiamine (*preT*), glyoxylate (*glcB* and *glcF*), and arabinose (*araA*), were similarly upregulated. Notably, we observed a significant upregulation of genes involved in nitrate assimilation (*narG*), cellular oxidant detoxification (*hcp*), as well as aerobic (*nuoM*) and anaerobic respiration (*dmsA*) in commensal *E. coli* co-cultured with SH (Fig. 4f).

#### Signal transduction and chemotaxis

Several genes involved in signaling and chemotaxis in commensal *E. coli*, especially five genes associated with bacterial phosphorelay signal transduction (*cheA, cheB, cadC, evgA,* and *norR*), four genes involved in chemotaxis and regulation of chemotaxis (*cheR, cheW, cheZ*, and *rbsB*), and one gene each for signal-complex assembly formation (*tar*), thermotaxis (*cheY*), and positive aerotaxis (*aer*), were significantly upregulated (Fig. 4g).

### Validation of gene expression data

Real-time quantitative PCR (RT-qPCR) was employed to validate the reliability and accuracy of RNA-sequencing data by quantifying relative expression patterns of six selected genes in SH and two genes in *E. coli* under the same co-culture conditions used in the transcriptome analysis experiment. Consistent with the RNA-seq analysis, RT-qPCR demonstrated a log_2_ fold-change (FC) expression of *sitA* (−0.9108), *punC* (−0.2713), *bsmA* (−0.8899), *mlrA* (−32.09), *sopE* (−0.117), and *bhsA* (2.1006) in SH and a log_2_ FC expression of *repHI2* (14.206) and *arcA* (3.499) in *E. coli*, thereby validating our transcriptomic profiling data.

## DISCUSSION

Our study focused on determining the differential gene expression in antibiotic-resistant SH in the presence of commensal *E. coli* to delineate the effect of commensal *E. coli* on fitness, virulence, and AMR in antibiotic-resistant NTS with a long-term goal of developing pre-harvest intervention strategies to reduce *Salmonella* colonization of poultry, thereby reducing foodborne salmonellosis in humans.

The results of gene enrichment analysis indicate the downregulation of genes involved in the formation of cell-division septum assembly in SH in the presence of commensal *E. coli*. The cell septum is a critical component for proper cell division and bacterial propagation in hosts (23). In this context, the downregulation of genes *zapA* and *zapB* in SH indicates a loose integration of the cell septum assembly. This leads to malfunctions or a complete absence of the z-ring protein (FtsZ), resulting in either a complete halt in the cell division or disruptions in the regulatory processes that initiate and complete bacterial cell division (24–26). Furthermore, the overexpression of genes involved in post-segregation killing of bacterial cells (*ccdA* and *ccdB*) in SH may be associated with impaired cell division in *Salmonella*, leading to low expressions of *zapA* and *zapB* genes. The differential expressions of these genes (*zapA*, *zapB*, *ccdA*, and *ccdB*) in SH18-9079 observed in this study could be critical for inducing cell death and reduced growth pattern of *Salmonella* in the presence of *E. coli* EC47-1826 (25, 27, 28). Additionally, reduced expression of cell division activator gene c*edA* and cell damage protection protein gene *nlpD* may further suppress cell division and growth of SH strain SH18-9079 in response to stress elicited by commensal *E. coli* strain EC47-1826 (29, 30). Therefore, the disruption of tightly and precisely controlled interplay of coordinated complex cellular processes by commensal *E. coli* involved in SH multiplication and growth may lead to reduced *Salmonella* burden in the chicken gastrointestinal tract, which is essential for its colonization and persistence in chickens, as well as for its transmission to new hosts (8, 23).

This study provided valuable insights into polyamine biosynthesis and putrescine catabolism, the pathways crucial for *Salmonella* virulence (31–33). Suppression of putrescine catabolism results in intracellular putrescine accumulation, critically inhibiting the synthesis of spermidine and spermine, which in turn may compromise the virulence, motility, cell surface adhesion, protein synthesis, and viability of *Salmonella* (32, 34–36, 37). In this study, the expressions of genes involved in putrescine biosynthesis from arginine (*speA*) or ornithine (*speF*) remained downregulated, while the expression of the *speE* involved in putrescine catabolism into spermidine was also downregulated (31, 33). The reduced expression of the compensatory putrescine transporter gene (*potF*) of SH may further diminish its virulent potential (38). Polyamine depletion affects the expression of genes associated with virulence, pathogenicity, and stress responses in *Salmonella*. Therefore, the downregulation of polyamine biosynthesis observed in this study could be critical to reduce the virulence and pathogenicity of SH by suppressing the T3SS (*sopE* and *sopF*) and the effector protein (*ssaD*) in SH (35). Therefore, the loss of polyamine biosynthesis and transport ability can substantially limit the pathogenicity of *Salmonella* by lowering the expression of virulence genes located on pathogenicity islands (32, 35). The lower expression of flagellar biosynthesis (*fhlE*) might diminish the motility of SH, which is essential for colonization and tissue invasion, thereby compromising *Salmonella* survival and virulence. Reduced *orgA* expression might further reduce SH mobility and invasiveness (32, 39, 40). Furthermore, the gene *fadR* involved in the biosynthesis of unsaturated fatty acids is vital to maintain a well-balanced ratio of unsaturated and saturated fatty acids for sustained biophysical properties of cell membrane phospholipids essential for bacterial growth and survival, probably through biofilm formation. Therefore, a significantly reduced expression of *fadR* might further compromise the growth and survival of *Salmonella* (41).

Likewise, enrichment analysis also provided considerable insights into metal ion acquisition, transportation, and utilization essential for the survival, pathogenicity, growth, and persistence of *Salmonella* inside the host (42–44, 45, 46). It has previously been reported that interactions with commensal *E. coli* suppress the expression of genes involved in metal ion homeostasis in *Salmonella* (20, 47). In this context, the downregulation of the inner membrane siderophore-independent system ABC-type transporter genes (*sitA*, *sitB, sitC*, and *sitD*) responsible for iron acquisition and transportation suggests reduced iron homeostasis in *Salmonella,* which is crucial for its virulence and persistence (20, 44). Furthermore, suppression of the transcriptional regulator of the xenosiderophore ferrioxamine transporter gene (*foxA*) in conjunction with the reduced expression of xenosiderophore-mediated iron uptake gene (*foxR*) might further diminish the iron uptake by *Salmonella* (48). Similarly, downregulation of *znuB*, *znuC*, *zniT,* and *zitB* would compromise the ability of *Salmonella* to maintain free intracellular zinc levels required for normal cellular homeostasis. The reduced expression of *znuB*, *znuC,* and *znuA* can also significantly impair the ability of *Salmonella* to adapt to its various environments and pathogenicity (42, 43). Moreover, suppression of genes responsible for copper and magnesium ion homeostasis may render a variety of enzymes critical for normal cellular function, cell growth, differentiation, and survival of *Salmonella* inactive (45, 46).

This study also sheds light on the expressions of genes essential for response regulation against reactive oxygen species (ROS). Lethal stress leads to intracellular accumulation of ROS and induces oxidative stress, damaging essential macromolecules. However, bacteria employ numerous defense proteins to detoxify ROS and counteract the damage incurred by ROS (49, 50). Therefore, the observed suppression of genes encoding superoxide dismutase (*sodC-2*) might subject SH to oxidative stress and impaired colonization (51, 52). In conjunction with this, suppression of another superoxide dismutase protein encoded by *sodA* may further increase the vulnerability of SH to ROS, leading to low growth and reduced survivability (53). Similarly, OmpX is critical in ROS stress, and therefore, the downregulation of *ompX* in the presence of commensal *E. coli* may enhance the vulnerability of SH to ROS stress and inhibit their growth (54). A notable reduction in the expressions of universal stress protein genes (*uspA, uspC,* and *uspE*) may further reduce the potential of *Salmonella* to withstand and survive under stressful conditions (55, 45). The ability of commensal *E. coli* to compete for luminal oxygen in the gastrointestinal tract also plays a role in reducing intestinal colonization of *Salmonella* (*18*).

ArcA, sometimes touted as a global regulator of gene expression, is an integral part of the two-component system ArcAB (anoxic redox control or aerobic respiration control) in *E. coli* (56, 57). Under anaerobic conditions, ArcA acts as a global response regulator controlling many operons and regulating diverse metabolic pathways either directly or indirectly (58). Therefore, ArcA plays a critical role in the survival and growth of *E. coli* in stressful and resource-competitive environments by regulating energy production via metabolic modifications, effective stress response, redox balance, and the acquisition and utilization of several key elements such as nitrogen, amino acids, and iron (56). In the current study, alterations in the transcriptomic profile of SH in co-culture may be linked to the observed upregulation of *arcA* in commensal *E. coli*. Under anaerobic conditions, *arcA* plays a critical role in balancing catabolic efficiency (energy production) and anabolic processes (biomass growth) through global regulation of metabolism. Our findings show a significantly higher growth of commensal *E. coli* in the co-culture along with a significant downregulation of genes critical for growth, persistence, and antimicrobial resistance dissemination observed in SH. This downregulation could be associated with the elevated expression of the *arcA* in the commensal *E. coli*, which might allow *E. coli* to competitively exclude SH from the chicken intestines (57). The expression of *arcA* in conjunction with other stress stimuli leads to metabolic reprogramming in *E. coli*, potentially enhancing the survival and growth of commensal *E. coli* under competitive conditions (56, 59). However, the connotation that the higher expression of *arcA* in commensal *E. coli* downregulated the expression of genes critical for colonization, pathogenicity, and survival in SH is speculative at this point, and further research is needed to substantiate such an association. Overall, the exact mechanism behind the transcriptomic shift that occurred in SH in the presence of a commensal *E. coli* strain remains unclear.

Commensal *E. coli* is one of the earliest colonizers of poultry intestines and predominates the gut microbiota population at a very young age (2, 10, 60). Sufficient intestinal colonization is essential for the effectiveness of probiotic bacteria. From this perspective, intestinal commensal *E. coli* has a competitive edge over other bacteria. The ability to naturally colonize chicken intestines exempts commensal *E. coli* from the multiple stresses encountered by other bacteria while passing through different intestinal segments to attain sufficient colonization in the chicken gut (8, 19). In addition, a significant downregulation of genes critical for metabolic and cellular pathways conferring fitness, virulence, and AMR dissemination potential provides evidence that commensal *E. coli* as a probiotic or its products as prebiotics can potentially be used to mitigate the impacts of drug-resistant SH and other NTS colonization and fecal shedding at the preharvest level in the poultry production systems (16, 20, 21). The selection of bacterial strains is a crucial aspect when studying microbial interactions. In this study, commensal *E. coli* strain EC47-1826 isolated from the cecum of a healthy broiler chicken was used as the representative commensal strain based on phylotyping results, confirming its classification under phylogroup B1, a group commonly associated with environmental and commensal *E. coli* rather than extraintestinal pathogenic *E. coli* (61). Studies have shown that phylogroups A and B1 are prevalent among *E. coli* strains isolated from healthy broilers, suggesting their role as commensals within the avian gut microbiome (62). The whole-genome sequencing and a revised avian *E. coli* pathotyping scheme further validated that EC47-1826 lacks potential virulence factors of avian pathogenic *E. coli* (63). This is particularly important in the context of microbial competition as the absence of virulence determinants proposed that the observed interactions of EC47-1826 with SH were not influenced by *E. coli* virulence determinants (64). Meanwhile, the SH18-9079 strain was chosen because it was a well-characterized ESBL-producing strain of SH isolated from the liver of a turkey with clinical salmonellosis (2).

This study offers valuable insights into the potential role of commensal *E. coli* in controlling NTS in poultry, as well as other animal species, at the farm level. However, the study has a few limitations. First, this study evaluated only one strain of poultry commensal *E. coli* and one strain of antibiotic-resistant SH; thus, the transcriptomic changes may not be valid for other strains of commensal *E. coli* and SH, and other NTS serovars. Second, since this study was an *in vitro* experiment, and therefore, the results need to be confirmed through *in vivo* experiments or field trials. Despite these limitations, the results of this study shed light on the behavior of SH in the presence of commensal *E. coli,* as revealed through differential transcriptomics profiling. This study also provides new insights into the potential ability of commensal *E. coli* to prevent colonization of NTS in poultry and offers the framework to explore the mechanisms by which commensal *E. coli* downregulates the expression of genes critical for fitness, virulence, and AMR potential in SH. Finally, this study highlights the promise of using species-specific commensal strains of *E. coli* to avert the colonization potential of *Salmonella* in poultry and possibly in other animal species and humans.

### Conclusion

Our research demonstrated that the commensal *E. coli* strain EC47-1826 suppresses the growth of the ESC-resistant SH strain SH18-9079. Additionally, it effectively downregulates the expressions of key genes responsible for critical metabolic pathways and cellular processes that drive the propagation, survival, pathogenicity and virulence, and AMR of SH. Specifically, the expressions of genes associated with cell proliferation, pathogenicity and virulence, biofilm formation, AMR and drug efflux, ion homeostasis and regulation, signal transduction and chemotaxis, stress response management, OMPs and transmembrane transportation, and cellular metabolism were significantly downregulated, whereas, in commensal *E. coli,* the genes involved in the cell proliferation, bacterial adherence and motility, ion homeostasis, signal transduction, chemotaxis, stress response to xenobiotics, OMPs and transmembrane transportation, and vital cellular metabolism were significantly upregulated. Notably, the upregulation of *arcA* might have enabled the commensal *E. coli* to effectively compete, survive, and grow in the co-culture with SH. Based on these compelling findings, we conclude that commensal *E. coli* significantly downregulates critical cellular pathways and functions that contribute to the fitness, virulence, and AMR to SH, which play a crucial role in the colonization of SH in the chicken intestines. Therefore, commensal *E. coli* or its products could be used as an alternative to antibiotics for controlling the growth of SH and potentially other NTS in poultry.

## MATERIALS AND METHODS

### Bacterial strains

The SH strain SH18-9079 isolated from the liver of a turkey with clinical salmonellosis, which is resistant to ESCs and harboring *bla*_CTX-M1_ (2), and the commensal *E. coli* strain EC47-1826 isolated from the cecum of a healthy broiler chicken were used in co-culture studies. To characterize EC47-1826 as a commensal *E. coli*, it was subjected to PCR-based phylogenetic typing (61) followed by the revised APEC pathotyping scheme (63) and whole-genome sequencing. Phylogenic typing grouped it as phylogroup B1 *E. coli*, whereas the APEC pathotyping scheme confirmed that it is a “no ST *E. coli.*” The chromosomal and plasmid sequences of EC47-1826 have been deposited in the National Center for Biotechnology Information (NCBI) Database under accession numbers CP184839 and CP184840, respectively.

### Bacterial culture

The SH and *E. coli* were co-cultured in Luria Bertani (LB) broth (BD, Difco, Franklin Lakes, NJ, USA) at an equal concentration (2.66 × 10^7^ CFU/mL, OD_600_ = 0.01) (JENWAY 7205 UV/Visible Spectrophotometer, Cole-Parmer Ltd, Staffordshire, UK) and incubated for 24 h at 37°C under anaerobic conditions, created with Mitsubishi AnaeroPack-Anaero System (Thermo Scientific, Swedesboro, NJ), with shaking. The cultures of SH and *E. coli* were also cultured separately under similar concentrations to serve as controls. Each bacterial culture had three biological replicates grown separately for bacterial colony counting and RNA extraction purposes.

### RNA extraction and rRNA depletion

Total RNA (RNA integrity number, RIN ≥ 8.5) was extracted using the RiboPure RNA Purification Kit (Invitrogen, Waltham, MA, USA) and further processed using a MICROBExpress Bacterial mRNA Enrichment Kit (Invitrogen, Waltham, MA, USA) to enrich for bacterial mRNA by depleting ribosomal RNA (rRNA) according to the manufacturer’s instructions. The quality and quantity of purified mRNA were determined using a NanoDrop 2000/2000c spectrophotometer (Thermo Fisher Scientific, Wilmington, DE, USA). Total RNA and mRNA quality were further assessed with 1% agarose and 1% denaturing formaldehyde gel electrophoresis, respectively.

### Library construction and RNA sequencing

Libraries were prepared using the TruSeq Stranded mRNA Library Kit (Illumina Inc., San Diego, CA, USA). The enriched mRNA was fragmented and primed to synthesize first- and second-strand cDNA. The double-stranded cDNA fragments were adenylated at the 3' end, ligated to multiple index adapters (TruSeq RNA Combinatorial Dual Indexes; Illumina Inc., San Diego, CA, USA), and enriched to amplify the amount of cDNA in the library. Finally, cDNA libraries were multiplexed and clustered in one lane of a flow cell for sequencing on a MiSeq System (Illumina, San Diego, CA, U.S.A.) with 2 × 300 bp paired-end (PE) reads and 100 M reads coverage. The SH and commensal *E. coli* grown separately at 37°C for 24 h in LB broth were used as controls to determine differential gene expressions. All sequences generated were deposited in the NCBI Gene Expression Omnibus (GEO) database under accession number GSE276976.

### Differential gene expression analysis

Quality assessment, base trimming, normalization, read mapping, and differential gene expression analysis were conducted through the CLC Genomics Workbench version 23.0.4 (QIAGEN Inc., Redwood City, CA, USA). The raw read sequences were imported into the CLC Genomics Workbench and aligned with SH chromosome (NCBI Refseq assembly: GCF_016452005.1) and its ESC-resistance encoding plasmid (NCBI Refseq assembly: NZ_MW349106.1) and *E. coli* APECO1 (NCBI Refseq assembly: GCF_000014845.1). Gene expressions were calculated using RPKM (reads/kilobase of exon model/million mapped reads) and applying the equation RPKM = number of gene reads/mapped reads (millions) × gene length (kb). Significant variations in the gene expression (upregulation or downregulation) were determined after employing the false discovery rate (FDR) (65). The TMM normalization (trimmed mean of M values) described for whole transcriptome RNAseq technology was applied to determine differential gene expression (DGE) in the two groups (SH +*E. coli* vs SH and SH +*E. coli* vs *E. coli*). The results of each bacterial culture grown in triplicate were averaged to determine the fold change (FC) in gene expression. Filtration criteria, such as FDR ≤ 0.05, FC ≥2.0, *P*-value ≤ 0.01, and FDR ≤ 0.05, FC <1.0, and *P*-value ≤ 0.01, were employed as cutoff values for DGE for upregulation and downregulation, respectively.

### Bacterial growth patterns

SH18-9079 and EC47-1826 were cultured separately and in co-culture at an equal concentration (OD_600_ = 0.01) in 50 mL tubes containing LB broth and incubated anaerobically at 37°C for 24 h with shaking. Subsequently, 100 µL volumes of ten-fold serial dilutions (10^−6^) of cultures were plated on McConkey agar. Each bacterial culture was plated in triplicates and incubated anaerobically at 37°C for 24 h. The number of bacteria or colony-forming units per mL (CFU/mL) was counted to determine the bacterial count.

### Validation of differential gene expressions

Relative gene expression results of SH18-9079 and commensal *E. coli* EC47-1826 grown in co-culture were validated by quantitative real-time PCR (qRT-PCR) in comparison to their respective controls. Six selected genes involved in metal-ion transport, iron acquisition and utilization (*sitA*), detoxification of xenobiotics (*punC*), bacterial survival and biofilm formation (*bsmA* and *bhsA*), motility (*mlrA*), and pathogenicity (*sopE*) in SH and two genes involved in replication (*repHI2*) and aerobic respiration control (*arcA*) in *E. coli* were used in qRT-PCR. The primers used are listed in Table S1 (Table S1). The 16S rRNA gene served as an endogenous control. Relative gene expression was determined according to the comparative critical threshold (Ct) method using QuantStudio Real-time PCR software V 1.5.2 (Applied Biosystems, Carlsbad, CA, USA). Finally, the data were normalized to the endogenous control 16S rRNA to determine the expression of selected genes in co-cultured bacteria compared to single bacterial cultures.

### Statistical analysis, software, and data preparation

For all quantitative assays, the three bacterial cultures mentioned above were grown separately in triplicates. Differential gene expressions were statistically analyzed using the CLC Genomics Workbench version 23.0.4 after applying the General Linear Model with the negative binomial distribution. A *P* ≤ 0.01 was considered statistically significant. Gene enrichment analysis was conducted in iDEP 2.0 (http://bioinformatics.sdstate.edu/idep/) to determine different biological pathways governed by significantly differentially expressed genes (SDEGs). Gene enrichment analysis plots were created using the SR plot (https://www.bioinformatics.com.cn/), whereas the heat maps were generated in the iDEP 2.0 and Morpheus (https://software.broadinstitute.org/morpheus/) by applying Euclidean distance with average linkage clustering for iDEP 2.0. Antibiotic-resistant genes were identified through the Comprehensive Antibiotic Resistance Database (CARD) (https://card.mcmaster.ca/), while virulence genes were identified using the Virulence Factor of Pathogenic Bacteria Database (VFDB) (http://www.mgc.ac.cn/VFs/). The paired *t*-test was used to analyze the viable bacterial colony counts of SH and *E. coli* in co-culture with bacterial colony counts of their respective cultures grown separately, whereas bacterial colony counts of SH and *E. coli* grown in co-cultures were analyzed by unpaired *t*-test in GraphPad Prism (10.2.3). A *P* ≤ 0.05 was considered significant.

## Data Availability

Transcriptomic profiles were deposited in the Gene Expression Omnibus (GEO) database in the NCBI under accession number GSE276976 (GEO Accession viewer). EC47-1826 whole-genome sequence is available from the NCBI under the accession numbers: chromosome - CP184839 (Escherichia coli strain 47-1826 chromosome, complete genome - Nucleotide - NCBI) and plasmid - CP184840 (Escherichia coli strain 47-1826 plasmid p47-1826, complete sequence - Nucleotide - NCBI).

## SUPPLEMENTAL MATERIAL

Table S1 spectrum.03336-24-s0001.csv

Table S2 spectrum.03336-24-s0002.csv Overall differential gene expression of *Salmonella* Heidelberg 189079.

Table S3 spectrum.03336-24-s0003.csv Overall differential gene expression of *E. coli* 471826.

ASM does not own the copyrights to Supplemental Material that may be linked to, or accessed through, an article. The authors have granted ASM a non-exclusive, world-wide license to publish the Supplemental Material files. Please contact the corresponding author directly for reuse.
